# Outcomes, risk factors and health burden of contrast-induced acute kidney injury: an observational study of one million hospitalizations with image-guided cardiovascular procedures

**DOI:** 10.1186/s12882-016-0385-5

**Published:** 2016-11-08

**Authors:** Pierre Aubry, Georges Brillet, Laura Catella, Aurélie Schmidt, Stève Bénard

**Affiliations:** 1 Hôpital Bichat-Claude Bernard, Paris, France; 2 Centre de néphrologie de Châteauroux, Châteauroux, France; 3 st[è]ve consultant, 30 rue Narcisse Bertholey, 69600 Oullins, France

**Keywords:** Contrast media, Cardiovascular procedure, Acute kidney injury, Epidemiology, Cost analysis

## Abstract

**Background:**

Despite the use of low-osmolar contrast media that have significantly reduced the occurrence of severe adverse reactions, contrast-induced (CI) acute kidney injury (AKI) remains the third cause of AKI in hospitals. We sought to estimate the frequency of CI-AKI among hospitalized patients undergoing image-guided cardiovascular procedures, to quantify the effect of risk factors on the development of this complication and to assess relative organizational and economic burden in healthcare.

**Methods:**

A retrospective cross-sectional population-based study using the extensive French hospital discharge database (PMSI) was carried out. Hospitalizations with image-guided cardiovascular procedures using a contrast media were identified in adults over a 2-year period (2012–2013). Suspected CI-AKI was defined as the presence, during hospitalization, of a diagnostic code of AKI (International Classification of Diseases, 10th revision [ICD-10] codes: N141, 142, N144, N990, N17, N19 or R392) or a code of renal replacement therapy procedure (Classification Commune des Actes Médicaux [CCAM] codes: JVJB001, JVJF002-005 and JVJF008) as creatinine criteria were not available.

**Results:**

During 1,047,329 hospitalizations studied, 32,308 suspected CI-AKI were observed, yielding a frequency of 3.1 %. By multivariate analysis, factors that significantly increased the risk of suspected CI-AKI included cardiogenic shock (odds ratio [OR] = 20.5, 95 % confidence interval [95 % CI] [18.7; 22.5]), acute heart failure (OR = 2.5, 95 % CI [2.4; 2.6]) and chronic kidney disease (OR = 2.3, 95 % CI [2.2; 2.3]. Renal replacement therapy was initiated during 6,335 (0.6 %) hospitalizations. The mean length of stay and cost of hospitalizations associated with suspected CI-AKI was higher than in hospitalizations without suspected CI-AKI (20.5 *vs* 4.7 days, *p* < 0.00001 and €15,765 *vs* €3,352, *p* < 0.0001, respectively).

**Conclusions:**

This is the first large-scale population-based study to estimate frequency and health burden of suspected CI-AKI occurring after image-guided cardiovascular procedures, and the first available data in a French population. We showed that this iatrogenic complication remains of high concern despite prevention efforts and contrast media product improvement. From our results, suspected CI-AKI is associated with particularly high mortality, significantly extends hospitalizations, and leads to additional costs reaching a total of €200M per year.

## Background

The use of iodinated contrast media (ICM) improves the visualization of blood vessels during image-guided procedures [1]. However, it may lead to iatrogenic renal injury, causing contrast-induced (CI) acute kidney injury (AKI).

AKI, formerly known as acute renal failure, is common in hospitalized patients and is strongly associated with morbidity and mortality. It is a complex syndrome that may arise in response to various aetiologies, such as the use of nephrotoxic drugs.

CI-AKI is the third most common cause of AKI in hospitals, after functional and medicine-related causes [2]. Depending on the definition, the population and the procedure studied, CI-AKI frequencies reported in the literature range from 1.5 to 15 % [3–7]. In most patients involved, serum creatinine returns to normal values within 2 weeks [8]. However, a subset of this population will need renal replacement therapy with associated increased hospitalization times and costs, and increased mortality [9, 10].

Many individual risk factors for the development of CI-AKI have been reported [9–15], predominant are chronic kidney disease, diabetes mellitus, congestive heart failure and age over 70 years.

The frequency of CI-AKI was expected to decrease with the use of low-osmolar ICM. In order to provide insights on the current state of this complication to health professionals and authorities, we conducted a cross-sectional retrospective population-based study using hospital data. The objective was to estimate the frequency of CI-AKI among hospitalized patients who underwent image-guided cardiovascular procedures, to quantify the effect of risk factors on CI-AKI occurrence and to assess the relative organizational and economic burdens.

## Methods

### Data source

Subjects were identified from the extensive French hospital discharge database (Programme de Médicalisation des Systèmes d’Information [PMSI]), which covers all French public and private hospitals involved in medicine, surgery and obstetrics. In 2004, French hospitals adopted a prospective payment system. Since then, the PMSI database has become the basis of hospital funding by third-party payers. A standard discharge summary report is generated for each hospitalization, and includes information on patient characteristics (e.g. sex, age, residence code), the main diagnosis that led to hospital admission, examinations carried out during hospitalization, comorbidities and possible complications. Biological data are not recorded. Diagnoses are coded using the International Classification of Diseases, 10th revision (ICD-10); procedures using the French classification of procedures in medicine (Classification Commune des Actes Médicaux [CCAM]). The discharge summary is then linked to a diagnosis-related group, used for the classification of hospitalizations, which are associated with national fees. Permission to extract and use the PMSI data was obtained from the National Commission for Data Protection and Liberties.

### Study population

The hospitalizations of adult patients (≥18 years) who had undergone image-guided cardiovascular interventions requiring intra-arterial or intra-venous ICM injections (Appendix 1) were extracted from 2012–2013 PMSI hospital databases. A committee of experts approved the selection of procedures.

Each hospitalization was considered as unique even though a patient could have had several different hospitalizations between January 1, 2012 and December 31, 2013.

### Identification of suspected CI-AKI

The primary endpoint was the onset of a suspected CI-AKI during a hospitalization, defined as the presence of (Appendix 2):

- a diagnosis of AKI (ICD-10 codes: N141, N142, N144, N990, N17, N19 or R392) or
- a renal replacement therapy procedure (CCAM codes: JVJB001, JVJF002, JVJF003, JVJF004, JVJF005 or JVJF008).

When renal replacement therapy was used during the hospitalization of interest, the suspected CI-AKI was classified as requiring renal replacement therapy; otherwise, it was considered as without renal replacement therapy.

End-stage kidney disease patients were identified by searching for a relevant diagnosis (ICD-10 code: N185) or a procedure of renal replacement therapy for chronic kidney disease (ICD-10 code: Z992; CCAM code: JVJF004, JVJF008 or JVJB001) within the 2 years prior to the hospitalization of interest. These hospitalizations were excluded from the analysis of suspected CI-AKI requiring renal replacement therapy as the renal replacement therapy would most likely have been related to the underlying disease.

### Collected data

All data were collected from the PMSI database. Known risk factors for developing CI-AKI (diabetes, chronic kidney disease, acute heart failure, onset of cardiogenic shock during the hospitalization) were recorded. The presence of diabetes (ICD-10 codes: E10-E14 or N083) or chronic kidney disease (ICD-10 code: N18) was searched for within the 2 years prior to the hospitalization, while acute heart failure (ICD-10 code: I50) and cardiogenic shock (ICD-10 code R570) were searched for only during the hospitalization.

In addition, sociodemographic characteristics (age and sex) and type of patient management (admission through the emergency department, hospitalization in an intensive care or resuscitation unit and hospital status) were collected.

### Statistical analysis

Quantitative data are expressed as means with standard deviations (SDs) or 95 % confidence intervals (95 % CIs); categorical data as numbers and percentages. Quantitative variables were compared using the Student’s *t*-test; categorical variables using the Chi-squared test.

Multivariable analysis using a mixed model with fixed effects (PROC GLIMMIX), taking into account the variability between hospitals, was performed to study suspected CI-AKI risk factors that required renal replacement therapy or not: gender, age, chronic kidney disease, diabetes, acute heart failure, cardiogenic shock, admission by the emergency unit and hospital status. Co-variables that were considered significant (*p* < 0.05) in suspected CI-AKI onset during bivariate analysis were included in the multivariable analysis.

Statistical analysis was performed using SAS 9.3® software (SAS Institute Inc. Cary, NC, USA). The significance level was fixed at 0.05 for all analyses.

### Economic analysis

The French Health Insurance perspective was used for the economic evaluation of hospitalizations. Each hospitalization is associated to a diagnosis-related group that is linked to a fixed tariff, which varies between public and private hospitals. Extra fees are added for hospitalizations with a visit to the intensive care unit, resuscitation unit, etc. All costs are expressed in 2015 euros. Costs incurred prior to this year were re-evaluated using the health service index (4011 E) (National Institute of Statistics and Economic Studies; Institut National de la Statistique et des Etudes Economiques [INSEE]).

## Results

### Hospitalization characteristics

During 2012–2013, a total of 1,047,329 hospitalizations involving an image-guided cardiovascular procedure requiring ICM administration was recorded in France. Hospitalizations most often included a coronary angiography procedure (45.4 %) and intraluminal dilation of one or several coronary vessels with endoprosthesis insertion (26.3 %). Procedures were not mutually exclusive. The most frequently reported reasons for hospitalization were cardiovascular events (Table 1).

**Table 1:** Characteristics of patients and procedures during hospitalizations

	Hospitalizations (*n* = 1,047,329)
Age (years), mean (SD)	66.1 (13.8)
Men, *n* (%)	712,586 (68.0 %)
Risk factors	
Diabetes	270,463 (25.8 %)
Chronic kidney disease	96,489 (9.2 %)
End-stage kidney disease	57,118 (5.5 %)
Acute heart failure	35,631 (3.4 %)
Cardiogenic shock	2,202 (0.2 %)
Procedures (>2 %)	
Coronary arteriogram	475,249 (45.4 %)
Intraluminal dilation of one or several coronary arteries with endoprosthesis insertion	275 812 (26.3 %)
Intraluminal dilation of one or several lower limbs artery with endoprosthesis insertion	54,420 (5.2 %)
Global arteriography of the abdominal aorta and lower limbs	43,599 (4.2 %)
Intraluminal dilation of internal iliac artery and/or external iliac artery with endoprosthesis insertion	32,631 (3.1 %)
Selective arteriography of 2 or more cervico-cephalic axes	28,388 (2.7 %)
Intraluminal dilation of one or more lower limbs artery without endoprosthesis insertion	25,875 (2.5 %)
Reasons for hospitalization (>5 %)	
Chronic ischaemic cardiomyopathy	189,474 (18.1 %)
Angina pectoris	165,852 (15.8 %)
Acute myocardial infarction	105,772 (10.1 %)
Arterial embolism and thrombosis	75,983 (7.3 %)
Atherosclerosis	71,147 (6.8 %)
Emergency department admission	151,826 (14.5 %)
Special unit admission	
Intensive care	263,236 (25.1 %)
Resuscitation	28,014 (3.6 %)
Deaths	19,624 (1.9 %)
Hospital status	
Private	442,957 (42.3 %)
Public	604,372 (57.7 %)

Data are presented as mean (SD) or *n* (%)

SD = standard deviation

Among all of the hospitalizations, the mean age was 66.1 years, and 68.0 % were male. Concerning risk factors, 25.8 % of hospitalizations involved patients with diabetes, 9.2 % with chronic kidney disease, 3.4 % with acute heart failure and cardiogenic shock occurred in 2,202 (0.2 %) of hospitalizations, among which 539 led to intra-aortic balloon pump use.

### Suspected contrast-induced acute kidney injury

#### General description

Suspected CI-AKI was found in 3.1 % (*n* = 32,308) of hospitalizations involving an image-guided cardiovascular procedure using ICM. Patients who developed suspected CI-AKI were older than those who did not (mean [SD]: 70.6 [13.6] *vs* 65.9 [13.8] years, *p* = 0.0017), but there was no significant gender difference (67.8 *vs* 68.0 % male, *p* = 0.30).

**Fig. 1:**
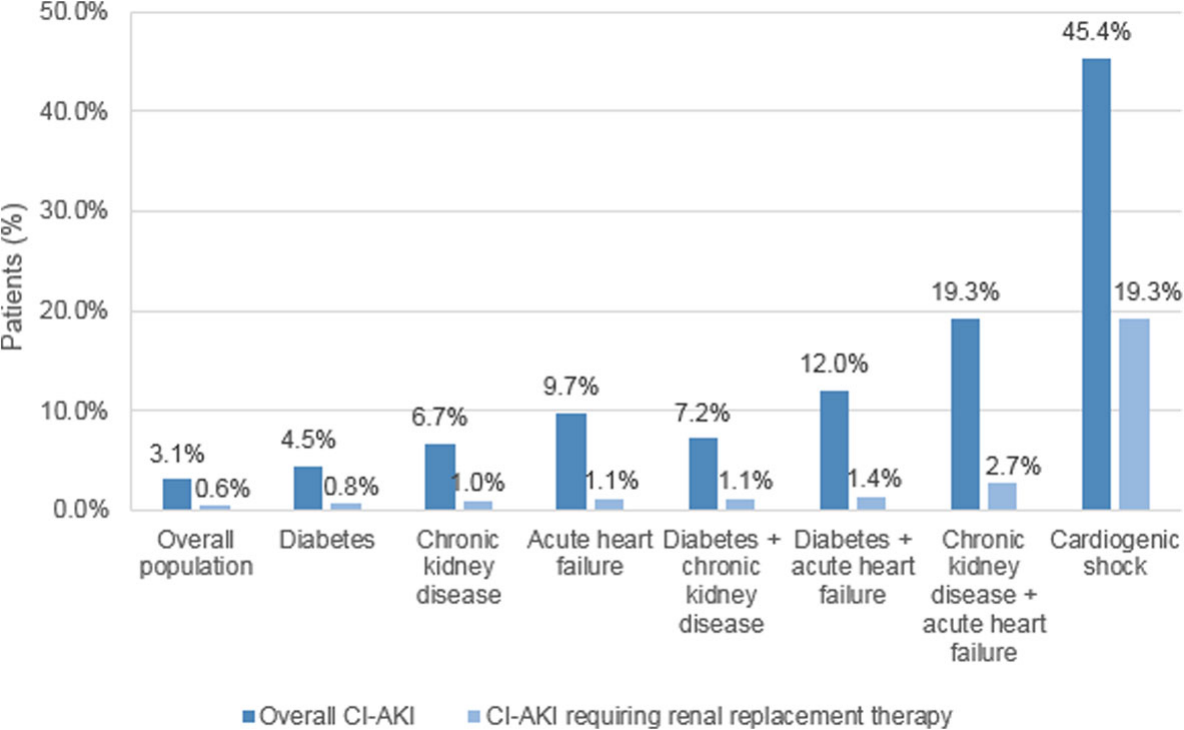
Proportions of hospitalizations with contrast-induced acute kidney injury, with and without renal replacement therapy. CI-AKI = contrast-induced acute kidney injury

The suspected CI-AKI proportion increased in hospitalizations involving patients with risk factors reaching 45.4 % (1,000/2,202) in hospitalizations during which patients presented with cardiogenic shock (Fig. 1).

**Fig. 2:**
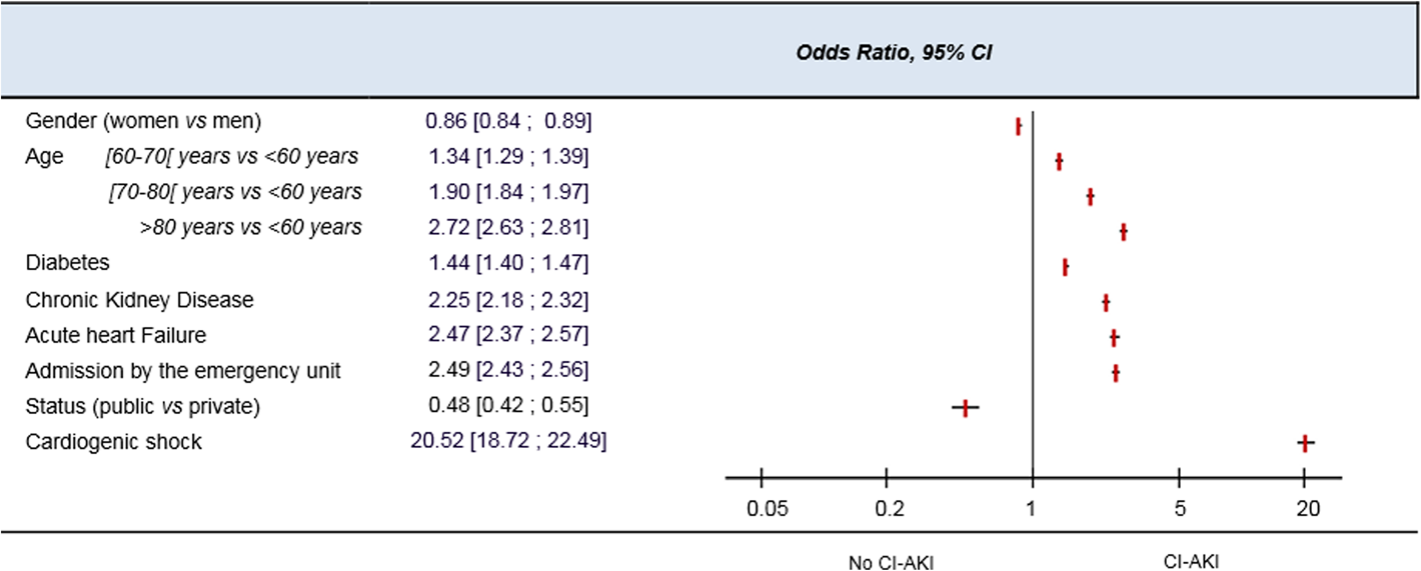
Risk factors for contrast-induced acute kidney injury onset. 95 % CI = 95 % confidence interval, CI-AKI = contrast-induced acute kidney injury

Multivariable analysis of suspected CI-AKI risk factors indicated that the occurrence of cardiogenic shock during a hospitalization gave the highest odds ratio (OR) for suspected CI-AKI onset (OR = 20.5, 95 % CI [18.7; 22.5]). Other risk factors with high ORs were: age > 80 (OR = 2.7 95 % CI [2.6; 2.8]), acute heart failure (OR = 2.5, 95 % CI [2.4; 2.6]), emergency admission (OR = 2.5, 95 % CI [2.4; 2.6]) and chronic kidney disease (OR = 2.3, 95 % CI [2.2; 2.3]; Fig. 2). All co-variables were significant in the bivariate analysis.

**Fig. 3:**
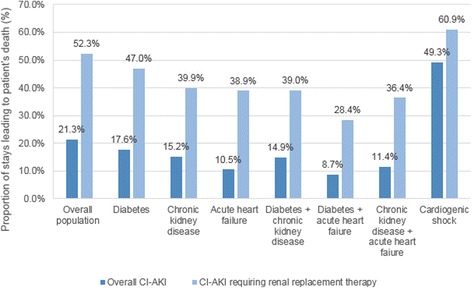
Proportion of stays leading to patient’s death associated with contrast-induced acute kidney injury, with and without renal replacement therapy. CI-AKI = contrast-induced acute kidney injury

Proportion of stays leading to patient’s death was 1.9 %, with a higher for suspected CI-AKI hospitalizations (21.3 % *vs* 1.3 for hospitalizations without suspected CI-AKI, *p* < 0.0001). Proportion of stays leading to patient’s death associated with suspected CI-AKI ranged from 8.7 to 49.3 % in hospitalizations involving patients who presented with risk factors (Fig. 3).

#### Requirement for renal replacement therapy

Among the total population, 0.6 % (*n* = 6,335) required renal replacement therapy. This was higher among hospitalizations of patients with risk factors (Fig. 1).

**Fig. 4:**
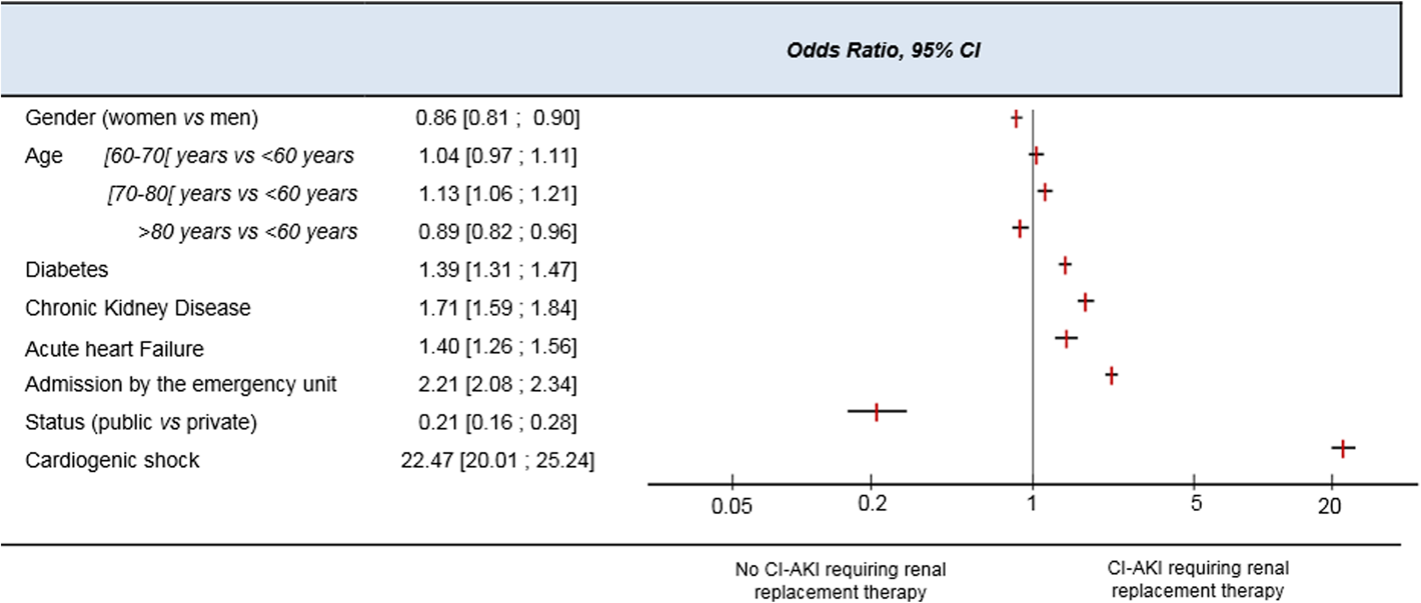
Risk factors for contrast-induced acute kidney injury requiring renal replacement therapy. 95 % CI = 95 % confidence interval

Multivariable analysis of risks factors for suspected CI-AKI requiring renal replacement therapy indicated that the occurrence of cardiogenic shock during a hospitalization also gave the highest OR for suspected CI-AKI requiring renal replacement therapy (OR = 22.5, 95 % CI [20.0; 25.2]). Other risk factors with high ORs were emergency admission (OR = 2.2, 95 % CI [2.1; 2.3]) and chronic kidney disease (OR = 1.7, 95 % CI [1.6; 1.8]; Fig. 4). All co-variables were significant in the bivariate analysis.

Hospitalizations during which suspected CI-AKI necessitated renal replacement therapy had a proportion of stays leading to patient’s death higher than hospitalizations with suspected CI-AKI and no renal replacement therapy (52.3 *vs* 13.7 %; *p* < 0.0001). Proportion of stays leading to patient’s during hospitalizations that involved patients with risk factors with suspected CI-AKI requiring renal replacement therapy ranged from 28.4 to 60.9 % (Fig. 3).

### Additional costs associated with suspected CI-AKI

#### Total population

**Fig. 5:**
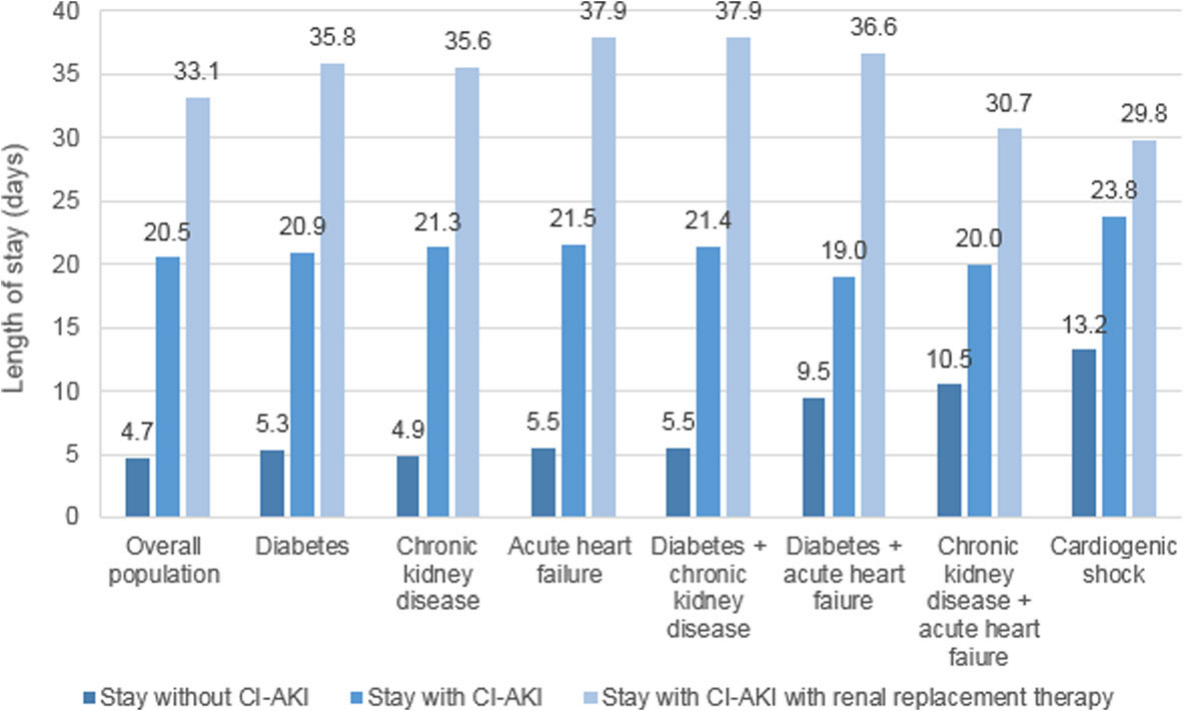
Lengths of stay associated with contrast-induced acute kidney injury, with and without renal replacement therapy. CI-AKI = contrast-induced acute kidney injury

**Fig. 6:**
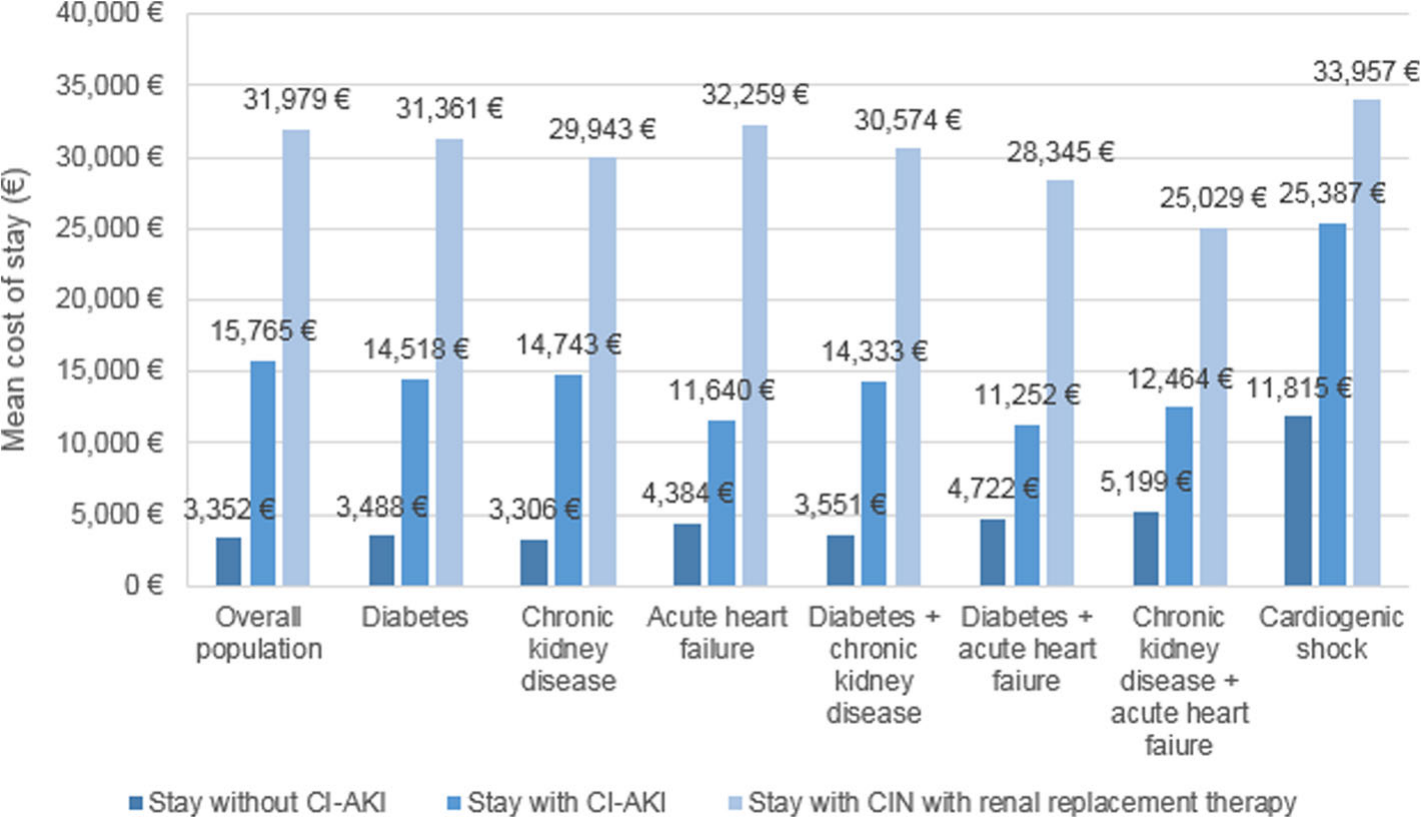
Costs of hospitalizations associated with contrast-induced acute kidney injury, with and without renal replacement therapy. CI-AKI = contrast-induced acute kidney injury

Suspected CI-AKI onset during a hospitalization led to a mean additional length of stay of 15.8 days (20.5 days, 95 % CI [20.2; 20.7] *vs* 4.7 days, 95 % CI [4.7; 4.7]; *p* < 0.00001; Fig. 5 and Table 2) and a mean extra cost of €12,413 (€15,765, 95 % CI [15,534; 15,995] *vs* €3,352, 95 % CI [3,343; 3,362]; *p* < 0.0001; Fig. 6 and Table 2).

**Table 2:** Durations and costs of hospitalizations with and without contrast-induced acute kidney injury

	Duration of hospitalization (days), mean [95 % CI]	Cost of hospitalization (Euros), mean [95 % CI]	Additional cost (Euros) for French Health Insurance over 2 years
Hospitalizations without suspected CI-AKI (*n* = 1,015,021)	4.7 [4.7; 4.7]	3,352 [3,343; 3,362]	-
Hospitalizations with suspected CI-AKI (*n* = 32,308)	20.5 [20.2; 20.7]	15,765 [15,534; 15,995]	401,023,050
*Requiring renal replacement therapy (n = 6,335)*	*33.1 [32.2; 34.0]*	*31,979 [31,181; 32,777]*	*181,348,244*

Data are presented as mean [95 % CI]

Costs are presented in 2015 Euro

*95 % CI* 95 % confidence interval, *CI-AKI* contrast-induced acute kidney injury

Additional lengths of stay and costs were higher for hospitalizations with suspected CI-AKI requiring renal replacement therapy than for hospitalizations without suspected CI-AKI, with a mean additional length of stay of 28.4 days (33.1 days, 95 % CI [32.2; 34.0] *vs* 4.7 days, 95 % CI [4.7; 4.7]; *p* < 0.00001; Fig. 5 and Table 2) and a mean additional cost of €28,627 (€31,979, 95 % CI [31,181; 32,777] *vs* €3,352, 95 % CI [3,343; 3,362]; *p* < 0.0001; Fig. 6 and Table 2). Overall, CI-AKI led to additional costs reaching a total of €401M over the 2-year period (Table 2).

#### Specific populations

Extra lengths of stay associated with suspected CI-AKI onset ranged from 9.5 to 16.4 days and from 20.2 to 32.4 days, depending on comorbidities, for hospitalizations of patients with suspected CI-AKI and suspected CI-AKI requiring renal replacement therapy, respectively (Fig. 5). Additional costs for these hospitalizations varied, respectively, from €6,530 to €11,437 and from €19,830 to €27,875, depending on comorbidities (Fig. 6).

Suspected CI-AKI onset led to an additional length of stay of 10.6 days and was associated with an extra cost of €13,572 for hospitalizations including patients with cardiogenic shock.

## Discussion

To the best of our knowledge, this is the largest study to estimate the frequency and health burden of CI-AKI occurring after image-guided cardiovascular interventions. Furthermore, this is the first available data on the general French population. Indeed, the few studies that have reported this complication in France focused on units treating severe cases, such as intensive care units [16, 17].

Using a national claims database to obtain extensive real-life data, we identified more than 1 million hospitalizations involving an image-guided cardiovascular procedure requiring ICM administration over a 2-year period. This data sample allowed us to obtain robust results in the absence of important data such as creatinine results.

The frequency of suspected CI-AKI was 3.1 % in our study for the 1,047,329 hospitalizations including an image-guide procedure using ICM, which represents a substantial proportion. The reported frequencies of suspected CI-AKI vary widely in the literature, ranging from 1.5 to 15 % [3–7], depending on the patient population and baseline risk factors. Furthermore, as with any clinical event, the frequency also varies depending on the criteria by which it is defined. Differences in the definitions used make it difficult to compare results of clinical studies (Table 3). CI-AKI is classically defined in the recent literature as a rise in serum creatinine occurring within the first 24 h after contrast exposure and peaking up to 5 days afterward. In most instances, the rise in serum creatinine is expressed either in absolute terms (0.5–1.0 mg/dL; 44.2–88.4 μmol/L) or as a proportional rise in serum creatinine of 25 or 50 % above the baseline value.

**Table 3:** Definitions of contrast-induced acute kidney injury

Association/Reference	Definition
European Society of Urogenital Radiology [26]	Increase in serum creatinine >0.5 mg/dL (44 μmol/L) or >25 % within 72 h
Mehran et al. [22]	Increase in serum creatinine ≥0.5 mg/dL (44 μmol/L) or ≥25 % within 48 h
Acute Kidney Injury Network [27]	Increase in serum creatinine ≥0.3 mg/dL (26 μmol/L) or ≥50 % or oliguria (<0.5 mL/kg/h for >6 h)
Kidney Disease Improving Global Outcomes [28]	Increase in serum creatinine of ≥0.5 mg/dL (44 μmol/L) or ≥25 %, assessed 48 h after a radiological procedure

*GFR* glomerular filtration rate

Renal replacement therapy was required in 0.6 % of hospitalizations, while the frequency of suspected CI-AKI requiring renal replacement therapy varies from 0.5 to 1 % in the literature [9, 18–20].

Our results come from a large and extensive database including all types of patients, with an extensive selection of image-guided cardiovascular procedures using ICM, thus allowing assessment of patients at all levels of severity.

The frequency of suspected CI-AKI was higher in hospitalizations involving patients with comorbidities (Fig. 1) and reached 45.4 % in hospitalizations with cardiogenic shock onset. This latter event was the greatest risk factor in our multivariable analysis with an OR of 20.5. Patients with chronic kidney disease or acute heart failure, and those who were admitted via emergency departments, had around twice the risk of developing CI-AKI.

CI-AKI was associated with extremely high in-hospital mortality of 21.3 %. The prognosis was even worse if the suspected CI-AKI required renal replacement therapy, with an in-hospital mortality reaching 52.3 %. Even though prior studies have reported mortality of 7 % for suspected CI-AKI without renal replacement therapy and 35 % for suspected CI-AKI requiring renal replacement therapy [9], but figures similar to ours have been reported in large retrospective cohort studies [10, 13, 21].

Surprisingly, mortality was lower during hospitalizations of patients with comorbidities than during those without (Fig. 3) despite a higher suspected CI-AKI frequency in these populations. One possible explanation for this lower mortality is the close monitoring of patients with important risk factors, leading to rapid detection and management of suspected CI-AKI. Furthermore, as it was not possible to study all of the confounding risks reported by Mehran et al., such as hypotension, contrast volume and haematocrit [22], the mortality results should be interpreted cautiously. Also, we could not analyse whether patients received non-steroidal anti-inflammatory drugs or were hyperglycaemic or volume depleted.

CI-AKI events were accompanied by an extreme increase in mean length of hospitalization (+15.8 days) and cost (+€12,413). The burden of this complication was higher when the suspected CI-AKI required renal replacement therapy, which increased the length of stay by 28.4 days and the cost by €28,627 compared with no CI-AKI. An economic analysis of the direct costs associated with CI-AKI has been conducted recently in the United States [23] – this study calculated the mean extra cost of CI-AKI onset to be $10,345 for the hospitalization in question and $11,812 for 1 year. The incidence data and results were calculated using a systematic search of the literature, and combined with unit costs, also obtained from the literature, using a decision-based analytical model. In our study, over the 2-year study period, it was estimated that onset of suspected CI-AKI during 1,047,329 hospitalizations led to additional overall expenditure for French Health Insurance of €401M.

Our study had several limitations, the majority of which are intrinsically linked to the PMSI database. This database is used in hospitals for budgetary reasons, and was not developed for epidemiological studies. Consequently, the data do not include results of clinical or biological testing. The absence of creatinine data represents the biggest limitation of our study. Therefore, to identify cases of suspected CI-AKI we selected, with the assistance of a Department of Medical Information doctor, the codes used in practice when this complication appears. Although the coding used to be poor in the early 2000’s, the quality has been widely improved in the past few years, showing high sensitivity and positive predictive value [24]. Furthermore, a selection of image-guided procedures usually requiring ICM administration was approved by a committee of experts. As the administration is not mandatory for all procedures selected and could not been verified, we conducted a secondary analysis on a restricted selection of image-guided cardiac procedures with mandatory use of ICM (DDQH and DGQH; Appendix 1). The results of this secondary analysis were similar to the main analysis and allowed us to validate the robustness of our results.

The PMSI database only allows observation of suspected CI-AKI with onset during hospitalization. However, CI-AKI onset can be up to 72 h after ICM administration and patients who are not considered at risk are often not hospitalized for such a long duration after the procedure. It is therefore possible that post-hospital events have not been taken into account. Nevertheless, as the mean length of hospitalization in this study was 4.7 days for patients without CI-AKI, we have assumed that the great majority of patients would still have been in hospital at the time of suspected CI-AKI onset, if it occurred.

Moreover, the PMSI database does not allow us to find the exact chronology of onset events during the hospitalization. Therefore, we have assumed that if, during a hospitalization that included a procedure requiring ICM administration, the patient presented with AKI, then the two events were related. However, we cannot state with certainty that all cases of AKI occurring in patients having ICM were attributed to CI-AKI. All cases of AKI, in the wider sense, have been taken into account and considered as suspected CI-AKI in this study. Nevertheless, some could be associated with cardiogenic shock or underlying conditions. This is, however, a limitation for most CI-AKI analyses as the accountability of ICM is often difficult to establish, even in clinical practice, as renal injury is multifactorial [25]. Furthermore, we observed cardiogenic shock in only 0.2 % of all hospitalizations, therefore, even if these events induced an AKI unrelated to ICM, it would not significantly impact our results. However, we note that we could not extract hypotension or use of potentially nephrotoxic agents (e.g. antibiotics or non-steroidal anti-inflammatory drugs) from the database, which are important potential causes of AKI.

## Conclusion

This is the largest population-based study that has estimated the frequency and health burden of suspected CI-AKI occurring after image-guided cardiovascular procedures. We have shown that this iatrogenic complication remains of high concern despite prevention efforts and contrast media product improvement. In this study, suspected CI-AKI was associated with particularly high mortality, significantly extended hospital length of stay, and additional costs reaching a total of €200M per year. Even though results should be interpreted cautiously due to limitations regarding absence of creatinine data and the impossibility to identify all risk factors of CI-AKI, we believe they are strengthened by the extremely large sample used. Besides, they are informative on the economic burden of CI-AKI when data are scarce.

## Key points

CI-AKI occurred in 3.1 % of image-guided cardiovascular procedures.

CI-AKI was associated with increased mortality.

CI-AKI was associated with significant hospital costs due to increased length of stay.

## Appendix 1

**Table 4:** Selected procedures (in the original French)

CCAM code	Detail of procedure
DAAF001	Dilatation intraluminale de la voie d'éjection du ventricule droit et du tronc de l'artère pulmonaire, par voie veineuse transcutanée
DAAF002	Dilatation intraluminale de la voie d'éjection infraaortique, par voie artérielle transcutanée
DAAF003	Agrandissement d'une communication interatriale, par voie veineuse transcutanée
DAGF001	Ablation de corps étranger intracardiaque ou intravasculaire, par voie vasculaire transcutanée
DAHF001	Biopsie de l'endocarde et du myocarde, par voie vasculaire transcutanée
DAMF001	Création d'une communication interatriale, par voie veineuse transcutanée
DASF001	Fermeture du conduit artériel, par voie vasculaire transcutanée
DASF002	Fermeture d'une déhiscence d'une prothèse de cloisonnement intraatrial, par voie vasculaire transcutanée
DASF003	Fermeture de communication interventriculaire, par voie veineuse transcutanée
DASF004	Fermeture d'une communication interatriale, par voie veineuse transcutanée
DASF005	Fermeture d'un foramen ovale perméable, par voie veineuse transcutanée
DBAF001	Dilatation intraluminale de l'orifice aortique, par voie artérielle transcutanée
DBAF002	Dilatation intraluminale de l'orifice pulmonaire sans perforation de la valve atrésique, par voie veineuse transcutanée
DBAF003	Dilatation intraluminale de l'orifice atrioventriculaire droit, par voie veineuse transcutanée
DBAF004	Dilatation intraluminale de l'orifice atrioventriculaire gauche, par voie veineuse transcutanée avec perforation du septum interatrial
DBAF005	Dilatation intraluminale de l'orifice pulmonaire avec perforation de la valve atrésique, par voie veineuse transcutanée
DBLF001	Pose d'une bioprothèse de la valve aortique, par voie artérielle transcutanée
DBLF009	Pose d'une bioprothèse de la valve pulmonaire dans un conduit prothétique, par voie veineuse transcutanée
DBSF001	Fermeture d'une déhiscence par désinsertion de prothèse orificielle cardiaque, par voie vasculaire transcutanée
DDAF001	Dilatation intraluminale d'un vaisseau coronaire sans pose d'endoprothèse, par voie artérielle transcutanée
DDAF003	Dilatation intraluminale de 3 vaisseaux coronaires ou plus avec pose d'endoprothèse, par voie artérielle transcutanée
DDAF004	Dilatation intraluminale de 2 vaisseaux coronaires avec pose d'endoprothèse, par voie artérielle transcutanée
DDAF006	Dilatation intraluminale d'un vaisseau coronaire avec pose d'endoprothèse, par voie artérielle transcutanée
DDAF007	Dilatation intraluminale de 2 vaisseaux coronaires avec artériographie coronaire, avec pose d'endoprothèse, par voie artérielle transcutanée
DDAF008	Dilatation intraluminale d'un vaisseau coronaire avec artériographie coronaire, avec pose d'endoprothèse, par voie artérielle transcutanée
DDAF009	Dilatation intraluminale de 3 vaisseaux coronaires ou plus avec artériographie coronaire, avec pose d'endoprothèse, par voie artérielle transcutanée
DDAF010	Dilatation intraluminale d'un vaisseau coronaire avec artériographie coronaire, sans pose d'endoprothèse, par voie artérielle transcutanée
DDFF001	Athérectomie intraluminale d'artère coronaire par méthode rotatoire [rotationnelle], par voie artérielle transcutanée
DDFF002	Athérectomie intraluminale d'artère coronaire, par voie artérielle transcutanée
DDPF002	Recanalisation d'artère coronaire avec pose d'endoprothèse, par voie artérielle transcutanée
DDQH006	Angiographie de pontage coronaire, par voie artérielle transcutanée
DDQH009	Artériographie coronaire sans ventriculographie gauche, par voie artérielle transcutanée
DDQH010	Artériographie coronaire avec ventriculographie gauche et artériographie thoracique [mammaire] interne unilatérale ou bilatérale, par voie artérielle transcutanée
DDQH011	Artériographie coronaire avec angiographie d'un pontage coronaire et ventriculographie gauche, par voie artérielle transcutanée
DDQH012	Artériographie coronaire avec ventriculographie gauche, par voie artérielle transcutanée
DDQH013	Artériographie coronaire avec angiographie de plusieurs pontages coronaires sans ventriculographie gauche, par voie artérielle transcutanée
DDQH014	Artériographie coronaire avec angiographie d'un pontage coronaire sans ventriculographie gauche, par voie artérielle transcutanée
DDQH015	Artériographie coronaire avec angiographie de plusieurs pontages coronaires et ventriculographie gauche, par voie artérielle transcutanée
DDSF001	Embolisation ou fermeture d'une fistule ou d'un anévrisme coronaire, par voie vasculaire transcutanée
DEEF001	Repositionnement de sonde définitive intracavitaire de stimulation cardiaque, par voie veineuse transcutanée
DEEF002	Repositionnement de sonde définitive intracavitaire de défibrillation cardiaque, par voie veineuse transcutanée
DFAF001	Dilatation intraluminale de plusieurs branches de l'artère pulmonaire avec pose d'endoprothèse, par voie veineuse transcutanée
DFAF002	Dilatation intraluminale d'une branche de l'artère pulmonaire sans pose d'endoprothèse, par voie veineuse transcutanée
DFAF003	Dilatation intraluminale d'une branche de l'artère pulmonaire avec pose d'endoprothèse, par voie veineuse transcutanée
DFAF004	Dilatation intraluminale de plusieurs branches de l'artère pulmonaire sans pose d'endoprothèse, par voie veineuse transcutanée
DFNF001	Fibrinolyse in situ de l'artère pulmonaire, par voie veineuse transcutanée
DFNF002	Thrombolyse mécanique ou thromboaspiration de l'artère pulmonaire, par voie veineuse transcutanée
DFQH001	Artériographie sélective du tronc et/ou des branches de l'artère pulmonaire, par voie veineuse transcutanée
DFQH002	Artériographie hypersélective des artères pulmonaires, par voie veineuse transcutanée
DFSF001	Oblitération d'anévrisme sacculaire de l'artère pulmonaire, par voie veineuse transcutanée
DFSF002	Embolisation de fistule artérioveineuse intrathoracique, par voie artérielle transcutanée
DGAF001	Dilatation intraluminale de l'aorte thoracique sans pose d'endoprothèse, par voie artérielle transcutanée
DGAF003	Dilatation intraluminale d'une coarctation de l'aorte abdominale, par voie artérielle transcutanée
DGAF004	Dilatation intraluminale d'une coarctation de l'aorte thoracique avec pose d'endoprothèse, par voie artérielle transcutanée
DGAF005	Dilatation intraluminale de l'aorte abdominale avec pose d'endoprothèse, par voie artérielle transcutanée
DGAF006	Dilatation intraluminale d'une coarctation de l'aorte thoracique sans pose d'endoprothèse, par voie artérielle transcutanée
DGAF007	Dilatation intraluminale de l'aorte thoracique avec pose d'endoprothèse, par voie artérielle transcutanée
DGAF008	Dilatation intraluminale de l'aorte abdominale sans pose d'endoprothèse, par voie artérielle transcutanée
DGLF001	Pose d'endoprothèse couverte bifurquée aortobisiliaque, par voie artérielle transcutanée
DGLF002	Pose d'endoprothèse couverte aorto-uniiliaque, par voie artérielle transcutanée
DGLF003	Pose d'endoprothèse couverte dans l'aorte thoracique, par voie artérielle transcutanée
DGLF005	Pose d'endoprothèse couverte rectiligne dans l'aorte abdominale infrarénale, par voie artérielle transcutanée
DGLF006	Pose d'un ballon de contrepulsion diastolique intraaortique, par voie artérielle transcutanée
DGLF012	Pose d’endoprothèse fenêtrée ou multibranche dans l’aorte abdominale pour anévrisme complexe, par voie artérielle transcutanée
DGPF001	Désobstruction de la bifurcation aortique, par voie artérielle transcutanée
DGPF002	Recanalisation de la bifurcation aortique avec pose d'endoprothèse, par voie artérielle transcutanée bilatérale
DGQH001	Artériographie globale de l'aorte abdominale et des membres inférieurs, par voie artérielle transcutanée
DGQH002	Artériographie globale de l'aorte abdominale, par voie artérielle transcutanée
DGQH003	Artériographie de l'aorte abdominale et des membres inférieurs, par injection intraaortique transcutanée lombale
DGQH004	Artériographie de l'aorte et de ses branches, par injection intraveineuse transcutanée
DGQH005	Artériographie globale de l'aorte thoracique et abdominale, par voie artérielle transcutanée
DGQH006	Artériographie globale de l'aorte thoracique, par voie artérielle transcutanée
DGQH007	Artériographie globale de la crosse de l'aorte et de ses branches cervicocéphaliques [Gerbe aortique], par voie artérielle transcutanée
DHAF001	Dilatation intraluminale de la veine cave supérieure avec pose d'endoprothèse, par voie veineuse transcutanée
DHAF002	Dilatation intraluminale de la veine cave inférieure sans pose d'endoprothèse, par voie veineuse transcutanée
DHAF003	Dilatation intraluminale de la veine cave supérieure sans pose d'endoprothèse, par voie veineuse transcutanée
DHAF004	Dilatation intraluminale de la veine cave inférieure avec pose d'endoprothèse, par voie veineuse transcutanée
DHGF001	Ablation d'un filtre temporaire de la veine cave inférieure, par voie veineuse transcutanée
DHNF001	Thrombolyse mécanique ou thromboaspiration de la veine cave supérieure, par voie veineuse transcutanée
DHNF002	Fibrinolyse in situ fémoro-ilio-cave, par voie veineuse transcutanée
DHNF003	Thrombolyse mécanique ou thromboaspiration de la veine cave inférieure, par voie veineuse transcutanée
DHNF004	Fibrinolyse in situ de la veine cave inférieure, par voie veineuse transcutanée
DHNF005	Fibrinolyse in situ de la veine cave supérieure, par voie veineuse transcutanée
DHNF006	Fibrinolyse in situ fémoro-ilio-cave avec oblitération partielle de la veine cave inférieure [pose d'un filtre cave], par voie veineuse transcutanée
DHPF001	Recanalisation de la veine cave supérieure sans pose d'endoprothèse, par voie veineuse transcutanée
DHPF002	Recanalisation de la veine cave supérieure avec pose d'endoprothèse, par voie veineuse transcutanée
DHPF003	Recanalisation de la veine cave inférieure sans pose d'endoprothèse, par voie veineuse transcutanée
DHQH001	Phlébographie sélective de plusieurs branches des veines iliaques communes et/ou de la veine cave inférieure, par voie veineuse transcutanée
DHQH002	Phlébographie de la veine cave inférieure [Cavographie inférieure], par voie veineuse transcutanée
DHQH003	Phlébographie de la veine cave supérieure [Cavographie supérieure], par injection intraveineuse transcutanée
DHQH004	Phlébographie sélective d'une branche de la veine iliaque commune ou de la veine cave inférieure, par voie veineuse transcutanée
DHQH005	Phlébographie des veines iliaque et cave inférieure [Iliocavographie], par injection intraveineuse transcutanée fémorale unilatérale ou bilatérale
DHQH006	Phlébographie globale de la veine cave supérieure [Cavographie supérieure], par voie veineuse transcutanée
DHQH007	Phlébographie hypersélective d'une branche de la veine iliaque commune ou de la veine cave inférieure, par voie veineuse transcutanée
DHSF001	Oblitération partielle temporaire de la veine cave inférieure, par voie veineuse transcutanée
DHSF002	Oblitération partielle définitive de la veine cave inférieure, par voie veineuse transcutanée
EAAF002	Dilatation intraluminale du tronc de l'artère carotide interne intracrânienne avec pose d'endoprothèse, par voie artérielle transcutanée
EAAF004	Dilatation intraluminale du tronc de l'artère carotide interne intracrânienne sans pose d'endoprothèse, par voie artérielle transcutanée
EAAF900	Dilatation intraluminale de branche de l'artère carotide interne avec pose d'endoprothèse, par voie artérielle transcutanée
EAAF901	Dilatation intraluminale de branche de l'artère carotide interne sans pose d'endoprothèse, par voie artérielle transcutanée
EAAF902	Dilatation intraluminale de l'artère vertébrale intracrânienne ou de l'artère basilaire avec pose d'endoprothèse, par voie artérielle transcutanée
EAAF903	Dilatation intraluminale de l'artère vertébrale intracrânienne ou de l'artère basilaire sans pose d'endoprothèse, par voie artérielle transcutanée
EACF001	Inversion du flux d'un anévrisme artériel intracrânien en période aigüe hémorragique, par voie artérielle transcutanée
EACF002	Inversion du flux d'un anévrisme artériel intracrânien en dehors d'une période aigüe hémorragique, par voie artérielle transcutanée
EANF002	Fibrinolyse in situ suprasélective d'artère intracrânienne, par voie artérielle transcutanée
EASF001	Oblitération de plusieurs anévrismes sacculaires artériels intracrâniens en dehors d'une période aigüe hémorragique, par voie artérielle transcutanée
EASF002	Occlusion intraluminale d'un vaisseau intracrânien afférent à une tumeur, par voie vasculaire transcutanée
EASF003	Occlusion intraluminale de plusieurs vaisseaux intracrâniens afférents à une tumeur, par voie vasculaire transcutanée
EASF004	Embolisation suprasélective unilatérale ou bilatérale de branche de l'artère carotide interne, par voie artérielle transcutanée
EASF005	Embolisation d'une fistule artérioveineuse durale cranioencéphalique multipédiculaire, par voie artérielle et par voie veineuse transcutanées
EASF006	Embolisation d'une fistule artérioveineuse durale cranioencéphalique unipédiculaire, par voie artérielle ou veineuse transcutanée
EASF007	Oblitération intraluminale d'une artère intracrânienne porteuse d'un anévrisme en période aigüe hémorragique, par voie artérielle transcutanée
EASF008	Oblitération intraluminale d'une artère intracrânienne porteuse d'un anévrisme en dehors d'une période aigüe hémorragique, par voie artérielle transcutanée
EASF009	Embolisation d'une fistule artérioveineuse durale cranioencéphalique unipédiculaire, par voie artérielle et par voie veineuse transcutanées
EASF010	Oblitération d'un anévrisme sacculaire artériel intracrânien en période aigüe hémorragique, par voie artérielle transcutanée
EASF011	Oblitération d'un anévrisme sacculaire artériel intracrânien en dehors d'une période aigüe hémorragique, par voie artérielle transcutanée
EASF012	Occlusion intraluminale d'un vaisseau intracrânien, par voie vasculaire transcutanée
EASF013	Oblitération de plusieurs anévrismes sacculaires artériels intracrâniens en période aigüe hémorragique, par voie artérielle transcutanée
EASF014	Embolisation sélective ou hypersélective unilatérale ou bilatérale de branche de l'artère carotide interne, par voie artérielle transcutanée
EASF015	Embolisation d'une fistule artérioveineuse durale cranioencéphalique multipédiculaire, par voie artérielle ou veineuse transcutanée
EBAF001	Dilatation intraluminale de l'artère carotide interne extracrânienne avec pose d'endoprothèse, par voie artérielle transcutanée
EBAF003	Dilatation intraluminale de l'artère carotide interne extracrânienne sans pose d'endoprothèse, par voie artérielle transcutanée
EBAF004	Dilatation intraluminale de l'artère carotide commune cervicale sans pose d'endoprothèse, par voie artérielle transcutanée
EBAF005	Dilatation intraluminale de l'artère carotide externe sans pose d'endoprothèse, par voie artérielle transcutanée
EBAF006	Dilatation intraluminale de l'artère carotide externe avec pose d'endoprothèse, par voie artérielle transcutanée
EBAF009	Dilatation intraluminale de la bifurcation carotidienne sans pose d'endoprothèse, par voie artérielle transcutanée
EBAF010	Dilatation intraluminale de l'artère carotide commune cervicale avec pose d'endoprothèse, par voie artérielle transcutanée
EBAF011	Dilatation intraluminale de la bifurcation carotidienne avec pose d'endoprothèse, par voie artérielle transcutanée
EBAF013	Dilatation intraluminale de l'artère vertébrale extracrânienne sans pose d'endoprothèse, par voie artérielle transcutanée
EBAF014	Dilatation intraluminale de l'artère vertébrale extracrânienne avec pose d'endoprothèse, par voie artérielle transcutanée
EBNF001	Fibrinolyse in situ sélective ou hypersélective d'une artère extracrânienne à destination cervicocéphalique, par voie artérielle transcutanée
EBNF002	Fibrinolyse in situ suprasélective d'une artère extracrânienne à destination cervicocéphalique, par voie artérielle transcutanée
EBQH001	Phlébographie globale d'un axe cervicocéphalique, par voie veineuse transcutanée
EBQH002	Artériographie sélective de 3 axes cervicocéphaliques ou plus, par voie artérielle transcutanée
EBQH005	Artériographie hypersélective cervicocéphalique, par voie artérielle transcutanée
EBQH007	Artériographie suprasélective cervicocéphalique, par voie artérielle transcutanée
EBQH008	Artériographie de plusieurs axes cervicocéphaliques, par injections intraartérielles transcutanées multiples
EBQH009	Phlébographie d'un axe cervicocéphalique, par injection intrajugulaire transcutanée
EBQH010	Artériographie d'un axe cervicocéphalique, par injection intraartérielle transcutanée unique
EBQH011	Artériographie sélective d'un ou 2 axes cervicocéphaliques, par voie artérielle transcutanée
EBSF001	Occlusion d'une fistule artérioveineuse directe cervicale ou crânienne, par voie vasculaire transcutanée
EBSF003	Embolisation sélective ou hypersélective unilatérale ou bilatérale de branche de l'artère carotide externe, par voie artérielle transcutanée
EBSF004	Embolisation suprasélective unilatérale ou bilatérale de branche de l'artère carotide externe, par voie artérielle transcutanée
ECAF001	Dilatation intraluminale d'une artère du membre supérieur avec pose d'endoprothèse, par voie artérielle transcutanée
ECAF002	Dilatation intraluminale d'une artère du membre supérieur sans pose d'endoprothèse, par voie artérielle transcutanée
ECAF003	Dilatation intraluminale du tronc artériel brachiocéphalique ou de l'artère carotide commune intrathoracique sans pose d'endoprothèse, par voie artérielle transcutanée
ECAF004	Dilatation intraluminale du tronc artériel brachiocéphalique ou de l'artère carotide commune intrathoracique avec pose d'endoprothèse, par voie artérielle transcutanée
ECJF001	Thromboaspiration d'artère ou de pontage artériel du membre supérieur, par voie artérielle transcutanée
ECLF003	Pose d'endoprothèse couverte dans une artère du membre supérieur, par voie artérielle transcutanée
ECLF004	Pose d'endoprothèse couverte dans le tronc artériel brachiocéphalique ou l'artère carotide commune intrathoracique, par voie artérielle transcutanée
ECLF005	Injection intraartérielle in situ sélective ou hypersélective d'agent pharmacologique anticancéreux au membre supérieur, par voie artérielle transcutanée
ECLF006	Injection intraartérielle in situ suprasélective d'agent pharmacologique anticancéreux au membre supérieur, par voie artérielle transcutanée
ECNF001	Fibrinolyse in situ suprasélective d'une artère ou d'un pontage artériel du membre supérieur, par voie artérielle transcutanée
ECNF002	Fibrinolyse in situ sélective ou hypersélective d'une artère ou d'un pontage artériel du membre supérieur, par voie artérielle transcutanée
ECPF001	Recanalisation d'une artère du membre supérieur avec pose d'endoprothèse couverte, par voie artérielle transcutanée
ECPF002	Recanalisation d'une artère du membre supérieur avec pose d'endoprothèse, par voie artérielle transcutanée
ECPF003	Recanalisation d'une artère du membre supérieur sans pose d'endoprothèse, par voie artérielle transcutanée
ECPF004	Recanalisation de l'artère subclavière en amont de l'ostium de l'artère vertébrale sans pose d'endoprothèse, par voie artérielle transcutanée
ECPF005	Recanalisation de l'artère subclavière en amont de l'ostium de l'artère vertébrale avec pose d'endoprothèse, par voie artérielle transcutanée
ECQH001	Artériographie bilatérale du membre supérieur par voie artérielle ou injection intraartérielle transcutanée, avec manœuvre positionnelle
ECQH002	Artériographie suprasélective du membre supérieur, par voie artérielle transcutanée
ECQH003	Artériographie unilatérale de la main, par injection intraartérielle transcutanée
ECQH004	Artériographie unilatérale du membre supérieur par voie artérielle ou injection intraartérielle transcutanée, avec manœuvre positionnelle
ECQH005	Artériographie sélective ou hypersélective du membre supérieur, par voie artérielle transcutanée
ECQH006	Artériographie du membre supérieur par injection intraartérielle transcutanée, sans manœuvre positionnelle
ECQH007	Artériographie bilatérale de la main, par injection intraartérielle transcutanée
ECQH012	Artériographie sélective ou hypersélective de la totalité de la moelle épinière, par voie artérielle transcutanée
ECQH013	Artériographie sélective ou hypersélective d'un segment de la moelle épinière, par voie artérielle transcutanée
ECQH014	Artériographie suprasélective de la moelle épinière, par voie artérielle transcutanée
ECQH015	Artériographie sélective ou hypersélective d'artère intrathoracique à destination pariétale et/ou viscérale, par voie artérielle transcutanée
ECQH016	Artériographie suprasélective d'artère intrathoracique à destination pariétale et/ou viscérale, par voie artérielle transcutanée
ECSF001	Oblitération de plusieurs anévrismes sacculaires artériels du membre supérieur, par voie artérielle transcutanée
ECSF002	Embolisation suprasélective d'une artère du membre supérieur, par voie artérielle transcutanée
ECSF003	Oblitération d'un anévrisme sacculaire artériel du membre supérieur, par voie artérielle transcutanée
ECSF004	Embolisation sélective ou hypersélective d'artère à destination bronchique ou pleuropulmonaire, par voie artérielle transcutanée
ECSF006	Embolisation suprasélective d'artère à destination bronchique ou pleuropulmonaire, par voie artérielle transcutanée
ECSF008	Embolisation sélective ou hypersélective d'une artère du membre supérieur, par voie artérielle transcutanée
EDAF001	Dilatation intraluminale sélective ou hypersélective de l'artère rénale avec pose d'endoprothèse, par voie artérielle transcutanée
EDAF002	Dilatation intraluminale de l'artère iliaque commune et/ou de l'artère iliaque externe sans pose d'endoprothèse, par voie artérielle transcutanée
EDAF003	Dilatation intraluminale de l'artère iliaque commune et/ou de l'artère iliaque externe avec pose d'endoprothèse, par voie artérielle transcutanée
EDAF004	Dilatation intraluminale de l'artère iliaque interne sans pose d'endoprothèse, par voie artérielle transcutanée
EDAF005	Dilatation intraluminale d'une artère digestive avec pose d'endoprothèse, par voie artérielle transcutanée
EDAF006	Dilatation intraluminale de l'artère iliaque interne avec pose d'endoprothèse, par voie artérielle transcutanée
EDAF007	Dilatation intraluminale sélective ou hypersélective de l'artère rénale sans pose d'endoprothèse, par voie artérielle transcutanée
EDAF008	Dilatation intraluminale d'une artère digestive sans pose d'endoprothèse, par voie artérielle transcutanée
EDAF009	Dilatation intraluminale suprasélective de l'artère rénale sans pose d'endoprothèse, par voie artérielle transcutanée
EDAF010	Dilatation intraluminale suprasélective de l'artère rénale avec pose d'endoprothèse, par voie artérielle transcutanée
EDJF001	Thromboaspiration de l'artère rénale, par voie artérielle transcutanée
EDJF002	Thromboaspiration d'artère digestive, par voie artérielle transcutanée
EDLF004	Pose d'endoprothèse couverte dans l'artère iliaque commune et/ou l'artère iliaque externe avec embolisation de l'artère iliaque interne, par voie artérielle transcutanée
EDLF005	Pose d'endoprothèse couverte dans l'artère iliaque commune et/ou l'artère iliaque externe, par voie artérielle transcutanée
EDLF006	Pose d'endoprothèse couverte dans une artère digestive, par voie artérielle transcutanée
EDLF007	Pose d'endoprothèse couverte dans l'artère iliaque interne ou une branche extradigestive de l'aorte abdominale, par voie artérielle transcutanée
EDLF008	Pose d'endoprothèse couverte dans plusieurs artères digestives, par voie artérielle transcutanée
EDLF013	Pose d'endoprothèse couverte dans l'artère rénale, par voie artérielle transcutanée
EDLF014	Injection intraartérielle hépatique in situ sélective ou hypersélective d'agent pharmacologique anticancéreux non radio-isotopique sans embolisation de particules, par voie artérielle transcutanée
EDLF015	Injection intraartérielle hépatique in situ suprasélective d'agent pharmacologique anticancéreux non radio-isotopique sans embolisation de particules, par voie artérielle transcutanée
EDLF016	Injection intraartérielle hépatique in situ suprasélective d'agent pharmacologique anticancéreux non radio-isotopique avec embolisation de particules, par voie artérielle transcutanée
EDLF017	Injection intraartérielle hépatique in situ sélective ou hypersélective d'agent pharmacologique anticancéreux non radio-isotopique avec embolisation de particules, par voie artérielle transcutanée
EDLF018	Injection intraartérielle rénale in situ suprasélective d'agent pharmacologique anticancéreux avec embolisation de particules, par voie artérielle transcutanée
EDLF019	Injection intraartérielle rénale in situ sélective ou hypersélective d'agent pharmacologique anticancéreux avec embolisation de particules, par voie artérielle transcutanée
EDLF020	Injection intraartérielle rénale in situ sélective ou hypersélective d'agent pharmacologique anticancéreux sans embolisation de particules, par voie artérielle transcutanée
EDLF021	Injection intraartérielle rénale in situ suprasélective d'agent pharmacologique anticancéreux sans embolisation de particules, par voie artérielle transcutanée
EDLL001	Injection intraartérielle hépatique in situ d'agent pharmacologique radio-isotopique avec embolisation de particules, par voie artérielle transcutanée
EDLL002	Injection intraartérielle hépatique in situ d'agent pharmacologique radio-isotopique sans embolisation de particules, par voie artérielle transcutanée
EDNF001	Fibrinolyse in situ suprasélective de l'artère rénale, par voie artérielle transcutanée
EDNF002	Fibrinolyse in situ sélective ou hypersélective de l'artère rénale, par voie artérielle transcutanée
EDNF003	Fibrinolyse in situ d'une artère digestive, par voie artérielle transcutanée
EDPF001	Recanalisation de l'artère iliaque interne avec pose d'endoprothèse, par voie artérielle transcutanée
EDPF002	Recanalisation de l'artère rénale sans pose d'endoprothèse, par voie artérielle transcutanée
EDPF003	Recanalisation d'une artère digestive sans pose d'endoprothèse, par voie artérielle transcutanée
EDPF004	Recanalisation d'une artère digestive avec pose d'endoprothèse, par voie artérielle transcutanée
EDPF005	Recanalisation de l'artère rénale avec pose d'endoprothèse, par voie artérielle transcutanée
EDPF006	Recanalisation de l'artère iliaque commune et/ou de l'artère iliaque externe avec pose d'endoprothèse couverte, par voie artérielle transcutanée
EDPF007	Recanalisation de l'artère iliaque interne sans pose d'endoprothèse, par voie artérielle transcutanée
EDPF008	Recanalisation de l'artère iliaque commune et/ou de l'artère iliaque externe sans pose d'endoprothèse, par voie artérielle transcutanée
EDPF009	Recanalisation de l'artère iliaque commune et/ou de l'artère iliaque externe avec pose d'endoprothèse, par voie artérielle transcutanée
EDQH001	Artériographie suprasélective de branche extradigestive de l'aorte abdominale ou de branche de l'artère iliaque interne, par voie artérielle transcutanée
EDQH003	Artériographie sélective ou hypersélective d'une branche extradigestive de l'aorte abdominale ou d'une branche de l'artère iliaque interne, par voie artérielle transcutanée
EDQH005	Artériographie sélective et/ou hypersélective de plusieurs branches extradigestives de l'aorte abdominale ou de plusieurs branches de l'artère iliaque interne, par voie artérielle transcutanée
EDQH006	Artériographie sélective et/ou hypersélective de plusieurs branches digestives de l'aorte abdominale, par voie artérielle transcutanée
EDQH007	Artériographie suprasélective de branche digestive de l'aorte abdominale, par voie artérielle transcutanée
EDQH008	Artériographie sélective ou hypersélective d'une branche digestive de l'aorte abdominale, par voie artérielle transcutanée
EDSF001	Oblitération suprasélective de plusieurs anévrismes sacculaires d'artère digestive, par voie artérielle transcutanée
EDSF002	Oblitération sélective ou hypersélective d'un anévrisme sacculaire de l'artère iliaque interne ou d'une branche extradigestive de l'aorte abdominale, par voie artérielle transcutanée
EDSF003	Embolisation sélective ou hypersélective de l'artère rénale, par voie artérielle transcutanée
EDSF004	Embolisation suprasélective de branche de l'artère iliaque interne ou de branche extradigestive de l'aorte abdominale, par voie artérielle transcutanée
EDSF005	Embolisation sélective ou hypersélective d'une artère digestive, par voie artérielle transcutanée
EDSF006	Embolisation suprasélective d'une artère digestive, par voie artérielle transcutanée
EDSF007	Oblitération sélective ou hypersélective d'un anévrisme sacculaire de l'artère rénale, par voie artérielle transcutanée
EDSF008	Embolisation suprasélective de l'artère rénale, par voie artérielle transcutanée
EDSF009	Oblitération suprasélective d'un anévrisme sacculaire d'une artère digestive, par voie artérielle transcutanée
EDSF010	Oblitération suprasélective d'un anévrisme sacculaire de l'artère iliaque interne ou d'une branche extradigestive de l'aorte abdominale, par voie artérielle transcutanée
EDSF011	Embolisation des artères iliaques internes [hypogastriques] et/ou de ses branches pour hémorragie du post-partum, par voie artérielle transcutanée
EDSF012	Embolisation sélective et/ou hypersélective de plusieurs artères digestives, par voie artérielle transcutanée
EDSF013	Oblitération suprasélective d'un anévrisme sacculaire de l'artère rénale, par voie artérielle transcutanée
EDSF014	Embolisation sélective et/ou hypersélective de plusieurs branches de l'artère iliaque interne ou de plusieurs branches extradigestives de l'aorte abdominale, par voie artérielle transcutanée
EDSF015	Embolisation suprasélective de plusieurs artères digestives, par voie artérielle transcutanée
EDSF016	Embolisation sélective ou hypersélective de l'artère iliaque interne ou d'une branche extradigestive de l'aorte abdominale, par voie artérielle transcutanée
EEAF001	Dilatation intraluminale de plusieurs artères du membre inférieur sans pose d'endoprothèse, par voie artérielle transcutanée
EEAF002	Dilatation intraluminale d'une artère du membre inférieur avec dilatation intraluminale de l'artère iliaque commune et/ou de l'artère iliaque externe homolatérale avec pose d'endoprothèse, par voie artérielle transcutanée
EEAF003	Dilatation intraluminale d'une artère du membre inférieur sans pose d'endoprothèse, par voie artérielle transcutanée
EEAF004	Dilatation intraluminale d'une artère du membre inférieur avec pose d'endoprothèse, par voie artérielle transcutanée
EEAF005	Dilatation intraluminale d'une artère du membre inférieur avec dilatation intraluminale de l'artère iliaque commune et/ou de l'artère iliaque externe homolatérale sans pose d'endoprothèse, par voie artérielle transcutanée
EEAF006	Dilatation intraluminale de plusieurs artères du membre inférieur avec pose d'endoprothèse, par voie artérielle transcutanée
EEJF001	Thromboaspiration d'artère ou de pontage artériel du membre inférieur, par voie artérielle transcutanée
EELF002	Pose d'endoprothèse couverte dans une artère du membre inférieur, par voie artérielle transcutanée
EELF004	Injection intraartérielle in situ suprasélective d'agent pharmacologique anticancéreux au membre inférieur, par voie artérielle transcutanée
EELF005	Injection intraartérielle in situ sélective ou hypersélective d'agent pharmacologique anticancéreux au membre inférieur, par voie artérielle transcutanée
EENF001	Fibrinolyse in situ suprasélective d'une artère ou d'un pontage artériel du membre inférieur, par voie artérielle transcutanée
EENF002	Fibrinolyse in situ sélective ou hypersélective d'une artère ou d'un pontage artériel du membre inférieur, par voie artérielle transcutanée
EEPF001	Recanalisation d'une artère du membre inférieur avec pose d'endoprothèse, par voie artérielle transcutanée
EEPF002	Recanalisation d'une artère du membre inférieur sans pose d'endoprothèse, par voie artérielle transcutanée
EEQH001	Artériographie bilatérale de membre inférieur, par injection intraartérielle fémorale transcutanée bilatérale
EEQH002	Artériographie sélective ou hypersélective du membre inférieur, par voie artérielle transcutanée
EEQH003	Artériographie du pied, par injection intraartérielle ou voie artérielle transcutanée
EEQH004	Artériographie suprasélective du membre inférieur, par voie artérielle transcutanée
EEQH005	Artériographie globale du membre inférieur, par voie artérielle transcutanée
EEQH006	Artériographie unilatérale du membre inférieur, par injection intraartérielle fémorale transcutanée
EESF001	Oblitération d'un anévrisme sacculaire artériel du membre inférieur, par voie artérielle transcutanée
EESF004	Embolisation hypersélective de plusieurs artères du membre inférieur, par voie artérielle transcutanée
EESF005	Oblitération de plusieurs anévrismes sacculaires artériels du membre inférieur, par voie artérielle transcutanée
EESF006	Embolisation suprasélective d'une artère du membre inférieur, par voie artérielle transcutanée
EESF007	Embolisation sélective ou hypersélective d'une artère du membre inférieur, par voie artérielle transcutanée
EFAF001	Dilatation intraluminale d'une veine du membre supérieur avec pose d'endoprothèse, par voie veineuse transcutanée
EFAF002	Dilatation intraluminale d'une veine du membre supérieur sans pose d'endoprothèse, par voie veineuse transcutanée
EFJF001	Thromboaspiration de la veine subclavière et/ou de la veine brachiocéphalique, par voie veineuse transcutanée
EFLF001	Pose d'endoprothèse couverte dans une veine du membre supérieur, par voie veineuse transcutanée
EFNF001	Fibrinolyse in situ de la veine subclavière et/ou de la veine brachiocéphalique, par voie veineuse transcutanée
EFPF001	Recanalisation d'une veine du membre supérieur avec pose d'endoprothèse, par voie veineuse transcutanée
EFPF002	Recanalisation d'une veine du membre supérieur sans pose d'endoprothèse, par voie veineuse transcutanée
EFQH001	Phlébographie sélective du membre supérieur par voie veineuse transcutanée, sans étude des troncs veineux proximaux
EFQH002	Phlébographie sélective de la veine brachiocéphalique ou de la veine cave supérieure, par voie veineuse transcutanée
EFQH003	Phlébographie bilatérale du membre supérieur par injection intraveineuse transcutanée, avec étude des troncs veineux proximaux et de la veine cave supérieure
EFQH004	Phlébographie bilatérale du membre supérieur par injection intraveineuse transcutanée, sans étude des troncs veineux proximaux
EFQH005	Phlébographie unilatérale du membre supérieur par injection intraveineuse ou voie veineuse transcutanée, avec étude des troncs veineux proximaux et de la veine cave supérieure
EFQH006	Phlébographie unilatérale du membre supérieur par injection intraveineuse transcutanée, sans étude des troncs veineux proximaux
EFQH007	Phlébographie hypersélective de la veine brachiocéphalique ou de la veine cave supérieure, par voie veineuse transcutanée
EGAF001	Dilatation intraluminale de la veine rénale sans pose d'endoprothèse, par voie veineuse transcutanée
EGAF002	Dilatation intraluminale de la veine iliaque externe et/ou de la veine iliaque commune avec pose d'endoprothèse, par voie veineuse transcutanée
EGAF003	Dilatation intraluminale de la veine iliaque externe et/ou de la veine iliaque commune sans pose d'endoprothèse, par voie veineuse transcutanée
EGAF004	Dilatation intraluminale de la veine rénale avec pose d'endoprothèse, par voie veineuse transcutanée
EGJF001	Thromboaspiration de la veine rénale, par voie veineuse transcutanée
EGJF002	Thromboaspiration de la veine iliaque externe et/ou de la veine iliaque commune, par voie veineuse transcutanée
EGNF001	Fibrinolyse in situ de la veine rénale, par voie veineuse transcutanée
EGNF002	Fibrinolyse in situ de la veine iliaque externe et/ou de la veine iliaque commune, par voie veineuse transcutanée
EGPF001	Recanalisation de la veine iliaque externe et/ou de la veine iliaque commune avec pose d'endoprothèse, par voie veineuse transcutanée
EGSF001	Embolisation suprasélective de la veine testiculaire ou ovarique, par voie veineuse transcutanée
EGSF002	Embolisation sélective ou hypersélective de la veine testiculaire ou ovarique, par voie veineuse transcutanée
EGSF003	Embolisation des veines de drainage du pénis, par injection intraveineuse transcutanée
EHAF001	Dilatation intraluminale de la veine porte ou d'une anastomose portocave avec pose d'endoprothèse, par voie veineuse transcutanée
EHAF002	Dilatation intraluminale de la veine porte ou d'une anastomose portocave sans pose d'endoprothèse, par voie veineuse transcutanée
EHAF003	Dilatation intraluminale de veine hépatique [veine sushépatique] sans pose d'endoprothèse, par voie veineuse transcutanée
EHAF004	Dilatation intraluminale d'une endoprothèse vasculaire intrahépatique pour court-circuit [shunt] vasculaire portosystémique, par voie veineuse transcutanée
EHCF002	Création d'un court-circuit [shunt] portosystémique intrahépatique par pose d'endoprothèse, par voie veineuse transcutanée
EHNF001	Fibrinolyse in situ de la veine porte et/ou de ses affluents ou d'un court-circuit [shunt] vasculaire portosystémique, par voie veineuse transcutanée
EHPF001	Recanalisation d'un court-circuit [shunt] vasculaire portosystémique, par voie veineuse transcutanée
EHQH001	Phlébographie sélective de veine hépatique [sushépatique], par voie veineuse transcutanée
EHSF001	Embolisation de varices œsogastriques ou des branches intrahépatiques de la veine porte, par voie veineuse transcutanée ou transpariétohépatique
EJQH001	Varicographie du membre inférieur, par injection intraveineuse transcutanée
EJQH002	Phlébographie bilatérale du membre inférieur par injection intraveineuse transcutanée au pied, avec iliocavographie par injection intraveineuse transcutanée fémorale bilatérale
EJQH003	Phlébographie rétrograde du membre inférieur, par injection intraveineuse transcutanée fémorale homolatérale ou par voie veineuse fémorale controlatérale
EJQH004	Phlébographie bilatérale du membre inférieur, par injection intraveineuse transcutanée au pied
EJQH005	Phlébographie rétrograde du membre inférieur, par injection intraveineuse transcutanée poplitée
EJQH006	Phlébographie unilatérale du membre inférieur, par injection intraveineuse transcutanée au pied
EJSF900	Occlusion de veine saphène par laser, par voie veineuse transcutanée
EJSF901	Occlusion de la grande veine saphène par radiofréquence, par voie veineuse transcutanée
EKQH002	Angiographie d'un accès vasculaire artérioveineux du membre supérieur avec exploration des troncs veineux profonds proximaux et de la veine cave supérieure, par injection intravasculaire transcutanée
ELSF001	Embolisation d'une fistule artérioveineuse rénale, par voie vasculaire transcutanée
ENAF001	Dilatation intraluminale d'un pontage artériel non anatomique des membres avec pose d'endoprothèse, par voie artérielle transcutanée
ENAF002	Dilatation intraluminale d'un pontage artériel non anatomique des membres sans pose d'endoprothèse, par voie artérielle transcutanée
ENFF001	Thrombectomie ou embolectomie mécanique d'un pontage artériel non anatomique des membres, par voie artérielle transcutanée
ENNF001	Fibrinolyse in situ d'un pontage artériel non anatomique des membres, par voie artérielle transcutanée
ENSF001	Embolisation d'une malformation vasculaire ou d'une lésion vertébrale, par voie vasculaire transcutanée
ENSF002	Embolisation de malformation artérioveineuse intraparenchymateuse de la moelle épinière, par voie vasculaire transcutanée
ENSF003	Embolisation de malformation artérioveineuse durale spinale, par voie vasculaire transcutanée
EQQH001	Mesure et enregistrement des pressions du cœur droit et de l'artère pulmonaire, avec injection de produit de contraste, par voie veineuse transcutanée
EQQH002	Mesure et enregistrement des pressions du cœur gauche et de l'aorte, avec injection de produit de contraste, par voie artérielle transcutanée
EQQH004	Mesure et enregistrement des pressions du cœur droit, de l'artère pulmonaire et du cœur gauche, avec injection de produit de contraste, par voie veineuse transcutanée avec perforation du septum interatrial
EQQH005	Mesure et enregistrement des pressions du cœur droit, de l'artère pulmonaire et du cœur gauche, avec injection de produit de contraste, par voie veineuse transcutanée et par voie artérielle transcutanée ou cathétérisme du foramen ovale, avant l'âge
EQQH006	Mesure et enregistrement des pressions du cœur droit, de l'artère pulmonaire et du cœur gauche, avec injection de produit de contraste, par voie veineuse transcutanée et par voie artérielle transcutanée ou cathétérisme du foramen ovale, à l'âge de
EZAF001	Dilatation intraluminale d'un accès vasculaire artérioveineux d'un membre sans pose d'endoprothèse, par voie vasculaire transcutanée
EZAF002	Dilatation intraluminale d'un accès vasculaire artérioveineux d'un membre avec pose d'endoprothèse, par voie vasculaire transcutanée
EZGF001	Ablation d'une endoprothèse vasculaire ou d'un filtre cave ayant migré en position intracardiaque ou intravasculaire, par voie vasculaire transcutanée
EZJF001	Thromboaspiration d'un accès vasculaire artérioveineux d'un membre avec dilatation intraluminale et pose d'endoprothèse, par voie vasculaire transcutanée
EZJF002	Thromboaspiration d'un accès vasculaire artérioveineux d'un membre avec dilatation intraluminale sans pose d'endoprothèse, par voie vasculaire transcutanée
EZNF001	Fibrinolyse in situ d'un accès vasculaire artérioveineux d'un membre avec dilatation intraluminale sans pose d'endoprothèse, par voie vasculaire transcutanée
EZNF002	Fibrinolyse in situ d'un accès vasculaire artérioveineux d'un membre avec dilatation intraluminale et pose d'endoprothèse, par voie vasculaire transcutanée
EZNF004	Fibrinolyse in situ d'un accès vasculaire artérioveineux des membres, par injection intravasculaire transcutanée
EZNF900	Irradiation intravasculaire, au décours d'une dilatation de vaisseau par voie vasculaire transcutanée
EZPF003	Désobstruction mécanique d'un accès vasculaire artérioveineux avec dilatation intraluminale et pose d'endoprothèse, par voie vasculaire transcutanée
EZPF004	Désobstruction mécanique d'un accès vasculaire artérioveineux avec dilatation intraluminale sans pose d'endoprothèse, par voie vasculaire transcutanée
EZQH002	Angiographie d'un accès vasculaire artérioveineux d'un membre, par voie vasculaire transcutanée
EZQH003	Angiographie d'un accès vasculaire artérioveineux d'un membre, par injection intravasculaire transcutanée

.

## Appendix 2

**Table 5:** Caption of codes used in methods (CCAM codes are in original French)

Classification	Codes	Caption
CIM-10	E10*	Insulin-dependent diabetes mellitus
	E11*	Non-insulin-dependent diabetes mellitus
	E12*	Malnutrition-related diabetes mellitus
	E13*	Other specified diabetes mellitus
	E14*	Unspecified diabetes mellitus
	I50*	Heart failure
	N08.3	Glomerular disorders in diabetes mellitus
	N14.1	Nephropathy induced by other drugs, medicaments and biological substances)
	N14.2	Nephropathy Induced By Unspecified Drug, Medicament Or Biological Substance
	N14.4	Toxic nephropathy, not elsewhere classified
	N18*	Chronic kidney disease
	N18.5	Chronic kidney disease, stage 5
	N99.0	Diagnosis - Postprocedural (Acute) (Chronic) Kidney Failure
	N17*	Acute Kidney Failure
	N19	Unspecified Acute Kidney Failure
	R39.2	Extrarenal uremia
	R57.0	Cardiogenic Shock
	Z99.2*	Dependence on renal dialysis
CCAM	JVJB001	Séance d'épuration extrarénale par dialyse péritonéale pour insuffisance rénale chronique
	JVJF002	Épuration extrarénale par hémodialyse, hémodiafiltration ou hémofiltration discontinue pour insuffisance rénale aigüe, par 24 heures
	JVJF003	Séance d'épuration extrarénale par hémoperfusion
	JVJF004	Séance d'épuration extrarénale par hémodialyse pour insuffisance rénale chronique
	JVJF005	Épuration extrarénale par hémodialyse, hémodiafiltration ou hémofiltration continue pour insuffisance rénale aigüe, par 24 heures
	JVJF008	Séance d'épuration extrarénale par hémodiafiltration, hémofiltration ou biofiltration sans acétate pour insuffisance rénale chronique

## Abbreviations

95 % CI: 95 % confidence interval
AKI: Acute kidney injury
CCAM: French classification of procedures in medicine (*Classification Commune des Actes Médicaux*)
CI: Contrast-induced
ICD-10: International Classification of Diseases, 10th revision
ICM: Iodinated contrast media
INSEE: National Institute of Statistics and Economic Studies (*Institut National de la Statistique et des Etudes Economiques*)
IQR: Interquartile range
OR: Odds ratio
PMSI: Programme for Medicalization of Information Systems (*Programme de Médicalisation du Système d’Information*)
SD: Standard deviation
WHO: World Health Organization
